# Exosomes as Pleiotropic Players in Pancreatic Cancer

**DOI:** 10.3390/biomedicines9030275

**Published:** 2021-03-09

**Authors:** Laura De Lellis, Rosalba Florio, Maria Cristina Di Bella, Davide Brocco, Francesca Guidotti, Nicola Tinari, Antonino Grassadonia, Rossano Lattanzio, Alessandro Cama, Serena Veschi

**Affiliations:** 1 Department of Pharmacy, “G. d’Annunzio” University of Chieti-Pescara, 66100 Chieti, Italy; laura.delellis@unich.it (L.D.L.); rosalba.florio@unich.it (R.F.); mariacristina.dibella@yahoo.it (M.C.D.B.); davide.brocco@unich.it (D.B.); francesca.guidotti@outlook.it (F.G.); 2 Department of Medical, Oral and Biotechnological Sciences, University “G. d’Annunzio”, Chieti-Pescara, 66100 Chieti, Italy; ntinari@unich.it (N.T.); grassa@unich.it (A.G.); 3 Center for Advanced Studies and Technology (C.A.S.T.), University “G. d’Annunzio”, Chieti-Pescara, 66100 Chieti, Italy; rossano.lattanzio@unich.it; 4 Department of Innovative Technologies in Medicine & Dentistry, University “G. d’Annunzio”, Chieti-Pescara, 66100 Chieti, Italy

**Keywords:** pancreatic cancer, extracellular vesicles, biomarkers, therapeutic targets, drug vehicles

## Abstract

Pancreatic cancer (PC) incidence is rising and due to late diagnosis, combined with unsatisfactory response to current therapeutic approaches, this tumor has an extremely high mortality rate. A better understanding of the mechanisms underlying pancreatic carcinogenesis is of paramount importance for rational diagnostic and therapeutic approaches. Multiple lines of evidence have showed that exosomes are actively involved in intercellular communication by transferring their cargos of bioactive molecules to recipient cells within the tumor microenvironment and systemically. Intriguingly, exosomes may exert both protumor and antitumor effects, supporting or hampering processes that play a role in the pathogenesis and progression of PC, including shifts in tumor metabolism, proliferation, invasion, metastasis, and chemoresistance. They also have a dual role in PC immunomodulation, exerting immunosuppressive or immune enhancement effects through several mechanisms. PC-derived exosomes also induce systemic metabolic alterations, leading to the onset of diabetes and weight loss. Moreover, exosomes have been described as promising diagnostic and prognostic biomarkers for PC. Their potential application in PC therapy as drug carriers and therapeutic targets is under investigation. In this review, we provide an overview of the multiple roles played by exosomes in PC biology through their specific cargo biomolecules and of their potential exploitation in early diagnosis and treatment of PC.

## 1. Introduction

Pancreatic cancer (PC) is considered an almost incurable disease, with 5-year survival barely reaching 10% [1]. Surgical resection is a potentially curative option for these patients, but approximately 80% of them present with advanced-stage unresectable disease at diagnosis [2]. Moreover, the 3-year recurrence rate exceeds 60% even when the most effective adjuvant chemotherapy regimens, such as mFOLFIRINOX, are used [3]. Therefore, for the majority of patients, chemotherapy represents the only available treatment, although results are largely unsatisfactory, with an overall survival of about 10 months [4]. Therapeutic strategies based on the use of natural compounds, novel synthetic molecules, or drug candidates for repurposing in oncology are also currently being explored, but these promising preclinical findings need additional evidence for translation into human therapy [5,6,7,8,9,10]. Unfortunately, PC appears to be a poorly immunogenic tumor characterized by the presence of a powerful immunosuppressive tumor microenvironment, which hinders substantial response to immunotherapy [11], whereas potential benefits of therapeutic strategies indicated in cases with specific molecular aberrations, such as defective mismatch repair or *BRCA1/BRCA2* mutations, are limited to small subgroups of patients [12]. Thus, a better understanding of the mechanisms responsible for refractoriness to chemo- and immuno-therapy is necessary to design more effective therapeutic strategies for PC treatment.

The communication between cancer and non-neoplastic cells has been recognized to play a crucial role in carcinogenesis, chemoresistance, and immunosuppression [13]. Intercellular communication is an essential hallmark of organized cells in multicellular organisms and is mediated through direct cell–cell contact or the transfer of functional biomolecules. Over the last decade, multiple lines of evidence have proposed extracellular vesicles (EVs) as key signal transducers in intercellular communication. EVs are a heterogeneous population of vesicles classified according to their origin, size, and properties [13]. They deliver specific biological information to recipient cells and have emerged as crucial regulators of organized cell communities in several physiological and pathological processes, including cancer [13]. EV-mediated intercellular communication occurs through different biological mechanisms of EV uptake and content release. Uptake may occur through several routes, including endocytosis, macropinocytosis, and phagocytosis [14,15]. Receptor-mediated endocytosis involves specific ligands on cancer-derived EVs’ membrane, which bind surface receptors on recipient cells to activate intracellular signaling [14]. Caveola- or clathrin-dependent and lipid raft-dependent endocytosis, together with direct fusion, are other distinct mechanisms of internalization that are independent of EV ligands [14]. Many types of cells release EVs, including dendritic cells (DCs), B and T cells, neurons, fibroblasts, stem cells, and cancer cells [16]. Because of their substantial stability EVs circulate systemically and have been detected in biological body fluids (i.e., plasma, urine, saliva, breast milk) and pathological effusions [17]. Moreover, EVs cross different biological barriers, as indicated by the presence of glial/neuronal EVs in the cerebrospinal fluid, blood, tears, and urine [13].

Exosomes, which are the focus of this review, are a small subtype of EVs characterized by high stability in extracellular fluids and circulation. They contain a repertoire of bioactive molecules that can be transferred locally and systemically [17]. Profiling of exosome cargo is a strategy for their characterization and for determining their cellular origin [18]. Specific delivery to distant targets is ensured by their peculiar surface molecules, which determine tropism to distinct cells and tissues [13]. The cargo carried by exosomes can be functionally exchanged between tumor and non-tumor cells to support key processes in cancer, including growth, angiogenesis, invasion, and pre-metastatic niche formation [19].

**Figure 1:**
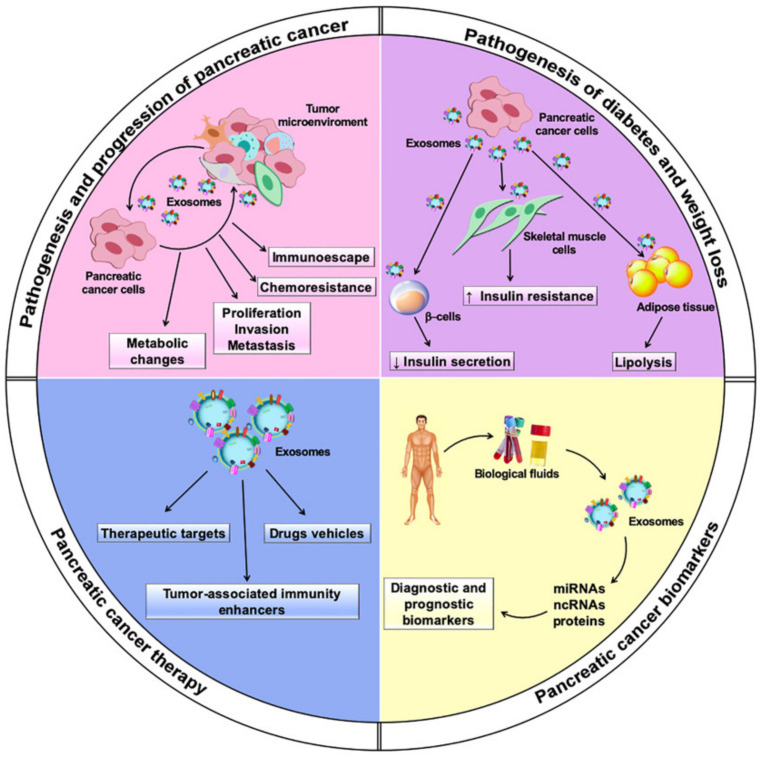
Multiple roles played by exosomes in pancreatic cancer. In the figure, the major topics discussed in the review are depicted.

In this review, we outline the multiple roles played by exosomes in PC biology through several functional cargo biomolecules, with a focus on their potential exploitation in PC diagnosis and therapy (Figure 1).

## 2. Exosomes Biogenesis and Secretion

**Figure 2:**
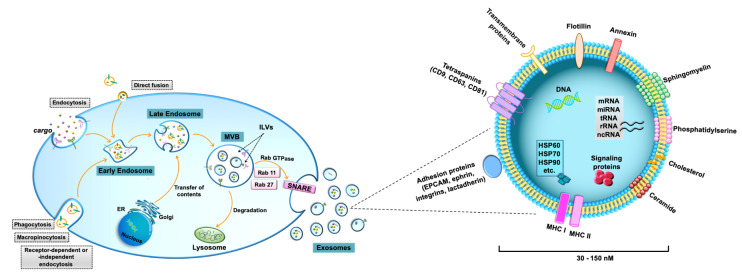
Biogenesis and composition of exosomes. Biogenesis of de novo exosomes starts from the formation of early endosomes by endocytosis of extracellular components. Early endosomes germinate inward, forming late endosomes and subsequently late endosomes mature as multivesicular bodies (MVBs) that contain intraluminal vesicles (ILVs). After fusion of MVBs with the plasma membrane, mature exosomes are released into the extracellular space by exocytosis. Exosome secretion is mediated by Rab GTPase proteins, such as Rab 11 and Rab 27, which control MVBs trafficking, as well as their binding to the plasma membrane and to soluble NSF-attachment protein receptor (SNARE) complexes. Additionally, recycled exosomes, which enter cells through several possible mechanisms, including macropinocytosis, phagocytosis, direct fusion, or caveola-, clathrin- or lipid raft-dependent endocytosis, participate in exosome assembly and cargo remodeling. Alternatively, both MVBs and recycled exosomes undergo fusion with lysosomes for degradation and release of their components into the cytosol. Exosomes are nano-sized vesicles, ranging from 30 to 150 nm. Their lipid bilayer membrane contains sphingomyelin, phosphatidylserine, cholesterol, and ceramide. Exosome surface proteins include specific tetraspanins (CD9, CD63, and CD81), adhesion proteins (e.g., EPCAM, ephrin, integrins, and lactadherin), growth factors, major histocompatibility complex (MHC) class I or class II molecules, annexin, and flotillin. Exosome cargo contains specific proteins, including cell signaling proteins, heat shock proteins (i.e., HSP60, HSP70, HSP90), and DNA and RNA molecules, such as microRNA (miRNA), messenger RNA (mRNA), transfer RNA (tRNA), ribosomal RNA (rRNA), and non-coding RNA (ncRNA).

The classification and nomenclature of EVs are continuously evolving [14,20]. Two major categories of EVs are commonly identified on the basis of the main EV biogenic pathways: ectosomes (microvesicles and oncosomes), which are generated by plasma membrane budding, and exosomes, which are formed by multivesicular body fusion with the plasma membrane [14,20]. In particular, exosomes are commonly distinguished by a series of biological features regarding size and composition [21]. Exosomes show diameters ranging from 30 to 150 nm and represent the smallest EVs (Figure 2) [14]. They consist of a lipid bilayer envelope surrounding a small cytosol devoid of cellular organelles [22]. The lipid bilayer includes sphingomyelin, phosphatidylserine, cholesterol, and ceramide and contains specific tetraspanins (CD9, CD63, and CD81), adhesion molecules (epithelial cell adhesion molecule-EPCAM, ephrin, integrins, and lactadherin), growth factors, and major histocompatibility complex (MHC) class I or class II molecules [21,23]. The expression of specific integrins and tetraspanins on the exosomal membrane regulates exosome binding/uptake and strongly influences target cell selection [22,24]. Exosomes also contain heat shock proteins (i.e., HSP60, HSP70, HSP90), annexins, flotillin, and other specific proteins, including cell signaling proteins [25]. Furthermore, exosome cargo contains RNA molecules, such as microRNA (miRNA), messenger RNA (mRNA), transfer RNA (tRNA), ribosomal RNA (rRNA), and non-coding RNA (ncRNA), as well as DNA, including mitochondrial DNA (mtDNA), single-stranded DNA (ssDNA), and double-stranded DNA (dsDNA) [26]. Some lipids, nucleic acids, and proteins are enriched in specific exosomes, whereas other proteins and lipids are present in all exosomes [21]. Exosomes are characterized by the presence of specific proteins, which reflect the parental cell [23].

The peculiar biogenesis of de novo exosomes starts from the formation of early endosomes by endocytosis of extracellular components (Figure 2) [15,21,27]. Early endosomes germinate inward, forming late endosomes and subsequently late endosomes mature as multivesicular bodies (MVBs) that contain intraluminal vesicles (ILVs). Independent pathways are involved in exosomal formation, including the endosomal sorting complex required for the transport (ESCRT)-dependent pathway and the ceramide-dependent pathway [16]. Protein sorting into ILVs is modulated by typical exosomal markers, such as the tetraspanins CD63 and CD81. Tetraspanin Tspan8 is also involved in the sorting of specific proteins and mRNA into ILVs. After fusion of MVBs with the plasma membrane, mature exosomes are released into the extracellular space by exocytosis [14,21] It is worth noting that also recycled exosomes, which enter cells through several possible mechanisms, including macropinocytosis, phagocytosis, direct fusion, or caveola-, clathrin-, or lipid raft-dependent endocytosis, can participate in exosome assembly, thus increasing exosome cargo complexity and heterogeneity (Figure 2).

Alternatively, both MVBs and recycled exosomes can be degraded by lysosomal hydrolases. Additionally, phagocytosis of opsin-enriched EVs by macrophages and other myeloid cell lineages leads to intracellular EV degradation [14]. Notably, sphingosine-1-phosphate (S1P) is a ceramide metabolite that plays a crucial role in the mechanisms related to exosome degradation into lysosomes, or exosome release [13]. In this regard, the inhibition of exosome secretion may increase MVB lysosomal degradation and vice versa [13].

Exosome secretion is mediated by Rab GTPase proteins, such as Rab 11 and Rab 27, which control MVBs’ trafficking, as well as their binding to the plasma membrane and to soluble NSF-attachment protein receptor (SNARE) complexes, allowing fusion of the lipid bilayer of MVBs and the plasma membrane [16,25]. Moreover, increasing intracellular Ca^2+^, lowering of pH in the microenvironment, and upregulation of p53 protein and of the enzyme heparanase stimulate exosome secretion [16]. It is worth noting that Bhattacharya et al. showed that GAIP interacting protein C terminus (GIPC) also regulates cellular trafficking pathways by modulating the secretion, biogenesis, and molecular composition of exosomes derived from PC cells [28]. Moreover, the GIPC status determines the loading of cellular cargo in exosomes [28]. Upon release into the extracellular space, the expression of membrane proteins, such as heparin sulphate proteoglycans and phosphatidylserine receptors, directs the interaction between exosomes and target cells [22].

Overall, the targeting of mechanisms involved in cancer exosome biogenesis and secretion may be potentially manipulated to hamper the communication between cancer cells and other recipient cells that are pivotal for cancer progression.

## 3. Role of Exosomes in the Pathogenesis and Progression of Pancreatic Cancer

**Figure 3:**
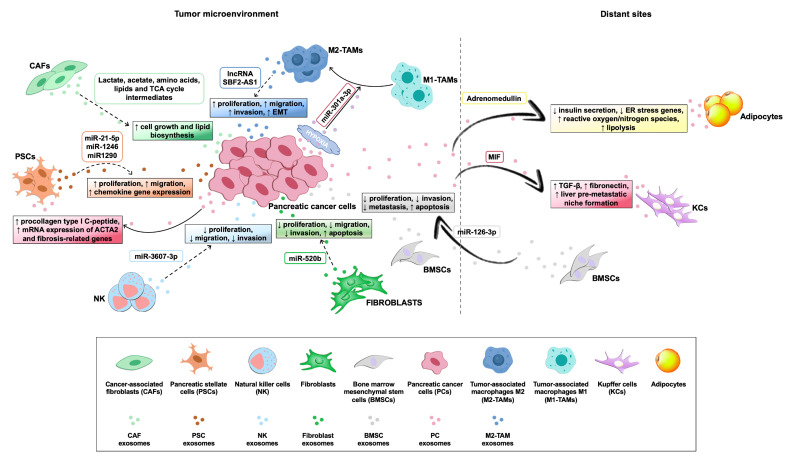
Roles of exosomes in the cross-talk among pancreatic cancer cells and cells within the tumor microenvironment, or at distant sites. Exosomes derived from pancreatic cancer (PC) cells and microenvironment cell components, together with their specific cargo molecules (rounded boxed text), play different roles (squared boxed text) in intercellular communication, by modulating metabolic activity and/or biological features of cells within the tumor microenvironment, or at distant sites. Solid arrows indicate the effects of PC cell-derived exosomes on recipient cells, whereas dashed arrows indicate the effects of microenvironment cell component-derived exosomes on PC cells.

Exosomes derived from tumor and non-tumor cells play a crucial role in distinct processes of PC biology, such as tumor–microenvironment communication and modulation of metabolic activity within the tumor microenvironment and at distant sites. In addition, exosomes exert their action in the progression, invasion, and metastasis of PC cells (Table 1), while also being involved in PC escape from immunosurveillance and induction of chemoresistance. Their role both in local and systemic cell–cell communication is exerted through the transfer of functional biomolecules, including miRNAs, other non-coding RNAs, and proteins, to recipient cells (Figure 3 and Table 1) [29].

**Table 1:** Roles of exosomes in the pathogenesis and progression of pancreatic cancer.

Role in PC	Exosome Source	Type of Study	Main Findings of the Study	Ref.
Metabolic changes, proliferation, invasion and metastasis	CAFs	In vitro	Inhibition of mitochondrial oxidative metabolism and promotion of glycolysis and glutamine-dependent reductive carboxylation in PC cells	[30]
	PSCs	In vitro	Stimulation of proliferation, migration and chemokine gene expression in PC cells	[31,32]
	PC cells	In vitro	Promotion of migration, invasion and EMT in PC cells by upregulation of miR-125b-5p and MEK2/ERK2 signaling	[33]
	NK cells	In vitro/ in vivo	Inhibition of PC malignant transformation by exosomal miR-3607-3p targeting of IL-26	[34]
	TAS	In vitro	Induction of PC cell apoptosis	[35]
	BMSCs	In vitro/ in vivo	Inhibition of PC cell proliferation, migration and invasion, together with induction of apoptosis in vitro and suppression of PC growth and metastasis in vivo by overexpression of exosomal miR-126-3p downregulating ADAM9	[36]
	PC cells	In vitro	Inhibition of PC cell proliferation through activation of pro-apoptotic phosphatase and GSK-3β; Notch-1 overexpression reverted PC cell proliferation inhibition	[37,38]
	Highly aggressive PC cells	In vitro/ in vivo	Promotion of PC cell migration and invasion via upregulation of CXCR4 and MMP-9 signaling; induction of tumor growth and liver metastasis formation in vivo	[39]
	Hypoxic PC cells	In vitro/ in vivo	Promotion of migration, invasion and EMT in PC cells through M2 polarization of macrophages induced by exosomal miR-301a-3p via PTEN/PI3Kγ pathway activation; high levels of miR-301a-3p facilitate lung metastasis in mice	[40]
	Highly aggressive PC cells	In vitro/ in vivo	Promotion of tumor invasion and metastasis in recipient PC cells by exosomal lnc-Sox2ot targeting of miR-200 family members	[41]
	Liver-metastatic PC cells	In vitro/ in vivo	Promotion of PC invasion by exosomal circ-PDE8A via MACC/MET/ERK and AKT pathway activation	[42]
	PC cells	In vitro/ in vivo	Alteration of endothelial permeability to promote invasion and metastasis by exosomal circ-IARS	[43]
	Highly aggressive PC cells	In vitro/ in vivo	Enhancement of PC cell proliferation, migration and invasion in vitro and promotion of PC growth in mice by upregulation of exosomal protein ZIP4	[44]
	PC cells	In vitro/ in vivo	Exosomal MIF-induced activation of human Kupffer cells, with increased TGF-β release and fibronectin production by hepatic stellate cells; induction of liver pre-metastatic niche	[45]
	PaCIC	In vitro	Stimulation of mobility, invasion and anchorage-independent growth of PC cells via CD44v6^+^ exosomes	[46]
	PC cells	In vitro/ in vivo	Promotion of matrix degradation, hematopoietic cell reprogramming towards an inflammatory phenotype, induction of protease- and chemokine- receptor expression in stromal cells and EMT in non-metastatic tumor cells by exosomal CD151 and Tspan8	[47]
Immunosuppression	Saliva from PC-bearing mice	In vitro/ in vivo	Ablation of immune NK cell cytotoxic potential against PC cells by salivary exosomes administered via gastrointestinal tract	[48]
	PC cells	In vitro	Suppression of TLR-4, TNF-α and IL-12 expression in dendritic cells through exosomal miR-203 overexpression	[49]
	PC cells	In vitro	Inhibition of RFXAP expression and subsequent impaired MHC-II expression in dendritic cells by exosomal miR-212-3p overexpression	[50]
	PC cells	In vitro	Induction of T lymphocytes apoptosis through p38 MAPK-mediated endoplasmic reticulum stress	[51]
Chemoresistance	GIPC-deficient PC cells	In vitro	Sensitization to gemcitabine of GIPC-depleted PC cells following the sequestering of the drug resistance gene *ABCG2* in secreted exosomes	[28]
	Gemcitabine-exposed CAFs	In vitro	Promotion of chemoresistance and PC cell proliferation by upregulating and delivering of Snail and of its downstream target miR-146a to recipient cells	[52]
	PC cells	In vitro	Upregulation of ROS detoxification enzymes CAT and SOD2; downregulation of gemcitabine-metabolizing gene DCK through exosomal miR-155	[53]
	PC cells	In vitro/ in vivo	Induction of anti-apoptotic activity in PC cells by exosomal miR-155 overexpression	[54]
	Gemcitabine-resistant PC cells	In vitro	Inhibition of cell cycle arrest and apoptosis induced by gemcitabine and promotion of cell migration by exosomal miR-210 delivery to gemcitabine-sensitive PC cells	[55]
	Gemcitabine-resistant PC cells	In vitro	Chemoresistance transmission to gemcitabine-sensitive PC cells by exosomal EphA2 overexpression	[56]

**Abbreviations:** Cancer-associated fibroblasts, CAFs; Pancreatic stellate cells, PSCs; Natural killer cells, NK; Pancreatic cancer, PC; Epithelial-mesenchymal transition, EMT; Interleukin 26, IL-26; Tumor-associated stroma TAS; Bone marrow mesenchymal stem cells, BMSCs; Metalloproteinase-9, ADAM9; Glycogen synthase kinase-3β, GSK-3β; C-X-C chemokine receptor type 4, CXCR4; Matrix metalloproteinase 9, MMP-9; Phosphatase and tensin homolog/phosphoinositide 3-kinase gamma, PTEN/PI3Kγ; Long non-coding RNA SOX2 overlapping transcript, lnc-Sox2ot; circular RNA PDE8A, circ-PDE8A; Circular RNA IARS, circ-IARS; Zinc transporter protein 4, ZIP4; Migration inhibitory factor, MIF; Transforming growth factor beta, TGF-β; Pancreatic cancer-initiating cells, PaCIC; CD44 variant isoform 6, CD44v6; Cluster of differentiation 151, CD151; Regulatory Factor X-Associated Protein, RFXAP; Major histocompatibility complex II, MHC-II; Mitogen-activated protein kinase, MAPK; GAIP interacting protein C terminus, GIPC; ATP-binding cassette sub-family G member 2 protein, ABCG2; Reactive oxygen species, ROS; Catalase, CAT; Superoxide dismutase 2, SOD2; Deoxycytidine kinase, DCK; Ephrin type-A receptor 2, EphA2.

### 3.1. Metabolic Changes, Proliferation, Invasion, and Metastasis

PC is characterized by a heterogeneous microenvironment including not only PC cells but also other components, including fibroblasts, pancreatic stellate cells (PSCs), extracellular matrix (ECM), tumor-associated macrophages (TAMs), immune cells, and adipocytes [57]. The interactions between tumor cells and the microenvironment, as well as with cells at distant sites induce changes in cancer cell metabolism and are pivotal in cancer progression and metastasis. In most cases, there is evidence for an important role for exosomes in these interactions (Table 1) [58,59,60].

Cancer-associated fibroblasts (CAFs) are the main cellular constituent of PC stroma and play a crucial role in tumor growth and invasion [61]. Zhao et al. found that CAF exosomes contain several metabolites, including lactate, acetate, amino acids, lipids, and tricarboxylic acid (TCA) cycle intermediates, which could be transferred to cancer cells to support their metabolic and synthetic needs [30]. In particular, CAF-derived exosomes inhibit mitochondrial oxidative metabolism, and promote glycolysis and glutamine-dependent reductive carboxylation. Consequently, there is a shift in cancer cell metabolism to favor cell growth and lipid biosynthesis [30]. Another study showed that exosomes released by gemcitabine-treated CAFs increase PC cell proliferation and survival, through overexpression of the transcription factor Snail and of its downstream target miR-146a [52].

PSCs are also key cellular components of the pancreatic tumor microenvironment and are known to contribute to tumor invasion and metastasis [31]. In healthy pancreatic tissue, PSCs are quiescent. In contrast, activated PSCs produce several types of inflammatory mediators, including interleukin 1 (IL-1), interleukin 6 (IL-6), interleukin 8 (IL-8), interleukin 15 (IL-15), connective tissue growth factor (cTGF), transforming growth factor β (TGFβ), and the CC-chemokine RANTES, which in turn activate other adjacent quiescent PSCs via an autocrine loop [62,63]. PSCs mediate microenvironment organization, closely interacting with different cell types, such as endothelial, neuronal, immune, and PC cells, through exosomal communication [32]. Interestingly, Masamune et al. found that PSC-derived exosomes stimulate proliferation, migration, and chemokine gene expression of PC cells, while the exosome release inhibitor GW4896 prevents these effects [32]. The cargo of PSC-derived exosomes is enriched in miR-21-5p, defined as an oncomiR, and also in miR-1246 and miR-1290. PSC-derived exosomes lead to increased PC cell proliferation and migration, and to induction of chemokine gene expression [31]. On the other hand, PC cell-derived exosomes induce activation and profibrogenic activities in PSCs [32]. In particular, PC cell-derived exosomes stimulate procollagen type I C-peptide production, as well as mRNA expression of α-smooth muscle actin (ACTA2) and fibrosis-related genes in PSCs, contributing to the development of a microenvironment suitable for cancer progression [32].

Natural killer (NK) cells present in the tumor microenvironment have been recognized to play a pivotal role in cancer progression [33,64]. Recently, Sun et al. co-cultured NK and PC cells to explore the potential involvement of cell-derived vesicles in affecting the proliferation, migration, and invasion of cancer cells [34]. In this context, NK cells are able to inhibit malignant transformation of co-cultured PC cells, both in vitro and in vivo, and miR-3607-3, which is enriched in NK cell exosomes, appears to play an important role in this process. Interestingly, miR-3607-3p appears to inhibit malignant transformation of PC cells through IL-26, one of its direct targets [34]. In line with the role of miR-3607-3p in PC malignant transformation, low levels of this miR predict poor prognosis in PC patients [34].

Additionally, adipocytes are known to play an important role in supporting cancer cells within the microenvironment of different tumors [65,66], and cancer cell-secreted exosomes were shown to have a crucial role in this interplay [67,68]. Cancer-associated adipocytes undergo metabolic changes, including delipidation, which supports cancer growth, as well as impaired glucose and lipid metabolism in co-culture with PC cells [69]. Although this has not been studied in detail, exosomes secreted by PC cells, in line with what has been observed with other cancers [67,68], are likely to play a relevant role in these phenotypic changes, since they were shown to induce lipolysis in co-cultured adipocytes [58].

The distinctive desmoplastic stroma in PC acts as a dynamic part in tumor cell proliferation and invasion (Figure 3 and Table 1) [70,71]. In addition to autocrine and paracrine interactions between CAFs and PC cells, it was shown that the catabolic phenotype observed in CAFs creates a nutrient-rich microenvironment and improves cancer cell metabolism, boosting the proliferation and development of metastasis [61]. Interestingly, several metabolic substrates are transferred from CAFs to tumor cells via exosomes [52]. In particular, CAF-derived exosomes are able to rescue the proliferation of nutrient-deprived BxPC3 and MiaPaCa-2 cells by providing them with metabolites in a KRAS-independent way [30].

Intriguingly, the paradoxical finding that strategies aimed at stromal depletion led to the progression of PC reinforced the need for further studies on the functional role of tumor-associated stroma (TAS) in the initiation and progression of PC [35]. Using co-cultured primary human pancreatic TAS cells and primary human xenograft-isolated PC cell lines it was shown that exosomes derived from human TAS cells played a tumor-suppressive role by inducing PC cell apoptosis [35]. Selective packaging of miRNAs into EVs led to the enrichment of stromal-specific miR-145 in exosomes released by TAS cells [35]. Notably, overexpressed miR-126-3p derived from bone marrow mesenchymal stem cell (BMSC) exosomes inhibited proliferation, invasion, and metastasis of PC cells through the downregulation of metalloproteinase-9 (ADAM9) [36]. BMSC-derived exosomes also promoted PC apoptosis both in vitro and in vivo [36]. Interestingly, exosomes secreted by human PC cell lines hamper the proliferation of PC recipient cells through the mitochondria-dependent apoptotic pathway, by activation of pro-apoptotic phosphatase and glycogen synthase kinase-3β (GSK-3β) [37]. Additional studies highlighted the essential role of Notch signaling in exosome-mediated apoptosis of PC cells. In particular, exosomal nanoparticles were shown to hamper the Notch-1 survival pathway, thereby activating the apoptotic pathway [38]. In a recent study, Shi et al. showed that miR-520b was poorly expressed in PC cells, but it was overexpressed in exosomes derived from normal fibroblasts [72]. Transfer of this fibroblast-derived miRNA via exosomes to PC cells induces silencing of its target zinc finger protein ZNF367 and suppresses PC cell proliferation, invasion, and migration, stimulating apoptosis [72]. Furthermore, in vivo experiments revealed that the overexpression of exosomal miR-520b inhibits tumor growth and liver metastasis, supporting the potential therapeutic role of this exosomal miRNA derived from normal fibroblasts in PC [72]. Remarkably, these in vitro and in vivo studies indicate that exosomes of different origins can exert opposite effects on PC cell proliferation.

Another study showed that exosomes derived from highly metastatic Panc02-H7 cells are able to decrease adhesion and increase migration and invasion of weakly metastatic Panc02 cells via CXCR4 and MMP-9 signaling [39]. In vivo, exosomes from metastatic Panc02-H7 cells induce liver pre-metastatic niche formation and foster both tumor growth and liver metastasis [39]. Recently, Wu et al. also explored the pro-metastatic role of exosomes derived from highly invasive PC cells in weakly invasive PC-1 cells [33]. The exosomal miRNA profile was analyzed, and miR-125b-5p was upregulated in highly invasive PC cells, increasing migration, invasion, and epithelial–mesenchymal transition (EMT). Moreover, its upregulation was associated with the activation of MEK2/ERK2 signaling [33]. Interestingly, a recent study showed that, in hypoxic conditions, PC-derived exosomes are involved in metastatic spread. In particular, hypoxic PC cell-derived exosomal miR-301a-3p promotes the polarization of M1 macrophages towards an M2 phenotype by activating the phosphatase and tensin homolog/phosphoinositide 3-kinase gamma (PTEN/PI3Kγ) pathway, which in turn stimulates migration, invasion, and EMT of PC cells [40]. In addition, circulating exosomal miR-301a-3p levels are positively associated with invasion, lymph node metastasis, late TNM stage, and poor prognosis in PC patients [40].

In addition to miRNAs, the role of other exosomal non-coding RNAs in PC progression was investigated. In particular, and lncRNA-Sox2ot from the exosomes of highly invasive PC cells was shown to promote in vitro and in vivo tumor invasion and metastasis in recipient PC cells through binding to miR-200 family members [41]. Moreover, high levels of exosomal Sox2ot in plasma are correlated with the TNM stage and overall survival of PC patients [41]. Notably, postoperative blood samples of PC patients with higher overall survival rates showed decreased exosomal Sox2ot expression, thus indicating that Sox2ot may represent a useful exosomal biomarker for PC progression or prognosis [41]. Recently, Yin et al. showed that lncRNA SBF2-AS1 derived from M2 macrophage exosomes also promotes the tumorigenicity of PANC-1 cells in nude mice [73]. This study revealed that the silencing of lncRNA SBF2-AS1 in M2 macrophage exosomes increased miR-122-5p expression to suppress X-linked inhibitor of apoptosis protein (XIAP) expression, which in turn inhibited PC progression [73]. Additionally, circular RNAs (circ-RNAs) contained in exosomes were recently shown to play relevant roles in different tumors [42]. A tumor-released circ-PDE8A derived from liver metastatic PC cells was identified both in plasma exosomes and in tumors from PC patients [42]. In particular, high circ-PDE8A expression correlates with lymphatic invasion, TNM stage, and poor overall survival in PC patients. Moreover, exosomal circ-PDE8A is likely to promote invasive growth of PC cells via the MACC/MET/ERK and AKT pathways [42]. Additionally, circular RNA IARS (circ-IARS) is overexpressed in PC tissues and in plasma exosomes of patients with metastatic disease. This circular RNA released by PC cells within exosomes regulates endothelial monolayer permeability, promoting tumor invasion and metastasis [43].

Several lines of evidence show that proteins carried by cancer cell-derived exosomes also promote cancer growth and progression. In this regard, a recent study showed that zinc transporter protein 4 (ZIP4), a membrane-localized zinc ion transporter, was the most upregulated exosomal protein in a highly malignant pancreatic cell line (PC-1.0). This protein plays an important role in boosting PC growth and interferes with gemcitabine uptake in PC cells through upregulation of ZEB1 and subsequent inhibition of the gemcitabine transporter ENT1 [74]. In vitro and in vivo studies showed that exosomal ZIP4 can significantly promote PC growth [44]. Moreover, a number of studies suggest that cancer cell-derived exosomes regulate PC metastasis by promoting the development of pre-metastatic niches in which they exert different effects, including inflammation, angiogenesis, matrix remodeling, and biomarker expression [19]. Costa-Silva et al. showed that PC-derived exosomes induce liver pre-metastatic niche formation in vivo and consequently increase liver metastatic spread [45]. Specifically, PC-derived exosomes enter the circulation and reach the liver, where they are incorporated by Kupffer cells, which in turn release TGF-β, promoting fibronectin production by hepatic stellate cells. Bone marrow-derived cells (i.e., macrophages and granulocytes) bind to fibronectin-enriched sites, contributing to liver pre-metastatic niche formation [45]. Moreover, they found that macrophage migration inhibitory factor (MIF) is overexpressed in PC-derived exosomes and its blockade counteracts liver pre-metastatic niche development. It is worth noting that MIF expression is higher in exosomes from stage I PC patients who later develop liver metastasis, indicating that exosomal MIF might be a prognostic biomarker of PC liver metastasis [45].

Additionally, exosomal proteins derived from cancer-initiating cells (CICs) are involved in metastatic spread. In this regard, Wang showed that CIC exosomes affect host cells by transferring CIC features into non-CIC cells [46]. In particular, CIC-derived exosomes are enriched in CD44 variant isoform v6 (CD44v6), a surface protein acting as a co-receptor for the receptor tyrosine kinase (RTK) MET and vascular endothelial growth factor receptor-2 (VEGFR-2). CD44v6 is upregulated in tumors with high metastatic potential and has a key role in supporting premetastatic niche formation via exosomes [75,76,77]. Specifically, exosomal CD44v6 promotes tumor progression by increasing mobility, invasion, and anchorage-independent growth of PC cells and is able to modulate the expression of the additional CIC marker tetraspanin Tspan8 in non-CIC cells [46]. In general, Tspan8, together with another tetraspanin CD151, act as metastasis-promoting proteins in different types of cancer [47]. Specifically, exosomal Tspan8 and CD151 were reported to increase the metastatic capacity of rat pancreatic adenocarcinoma ASML cells [47]. By comparing a wild-type ASML cell line with CD151-knockdown and/or Tspan8-knockdown clones, Yue et al. showed that tumor-derived exosomal proteins Tspan8 and CD151 are involved both in promoting ECM degradation, through the activation of associated proteases and integrins, as well as in reprogramming hematopoietic cells towards an inflammatory phenotype, by inducing the overexpression of chemokine/chemokine receptors in stroma cells [47] Moreover, CD151/Tspan8-competent tumor exosomes promote EMT in non-metastatic cells [47]. Thus, the depletion of exosomal tetraspanins Tspan8 and CD151 represents a potential and interesting strategy to decrease the metastatic potential of PC [47].

In summary, all these findings indicate that exosomes derived from PC cells and other cells within the PC microenvironment play a key role in proliferation, invasion, and metastasis, and in the modulation of metabolism through several functional biomolecules, also acting as mediators in the preparation of distant sites for pre-metastatic niche formation.

### 3.2. PC Escape from Immunosurveillance

Several studies focused on the immunosuppressive and pro-metastatic effects that PC exosomes exert within the tumor microenvironment (Table 1). In general, the tumor microenvironment is characterized by inadequate immune surveillance and tolerance to tumor cells. Exosomes have a dual role by exerting an immunosuppressive effect but also by triggering an anticancer response through the presentation of tumor antigens to dendritic cells [78]. Several in vivo and in vitro experiments indicated that tumor-associated exosomes affect immune surveillance both in the tumor microenvironment and at distal cell targets. They can hamper maturation of dendritic cells, impair NK cell activation, and skew myeloid-derived suppressor cells and macrophages towards a tumorigenic phenotype. Exosomes can also promote effector T cell apoptosis via Fas/FasL interaction and foster regulatory T cell proliferation by releasing the inhibitory cytokine TGF-β [79].

Exosomes are detected in all body fluids, including urine, tears, and saliva. Katsiougiannis et al. showed that saliva from mice with PC, when administered by oral gavage to healthy mice, impairs the activation of peripheral NK cells in these animals [48]. Moreover, oral administration of salivary exosomes from tumor-bearing mice is able to transfer the inhibition of NK cytotoxic activity against PC cells to other mice [48]. Specifically, the authors showed that exosomes, abundantly detected in mouse saliva, act as mediators of this mechanism. They suggest that tumor-derived exosomes are shuttles that travel systemically and cause a remodeling of salivary gland exosomes, which in turn are able to affect the cytotoxic potential of NK cells in PC. Moreover, salivary exosomes from PC-bearing mice in which pancreatic tumors are engineered to suppress exosome biogenesis fail to ablate NK cells’ cytotoxic potential against PC cells, as compared to salivary exosomes from PC-bearing mice with normal tumor exosome biogenesis. Thus, in addition to their role as biomarker carriers, salivary exosomes are also likely to exert a downstream detrimental effect upon peripheral NK cells via the gastrointestinal tract [48].

It is widely accepted that the ability of PC cells to elude immunosurveillance is due, at least in part, to miRNAs that can be delivered by tumor-derived exosomes into immune cells, causing inhibition of mRNA expression and thus affecting the immune response against tumor cells [49]. Some miRNAs are enriched in PC-derived exosomes and are able to affect mRNA expression of dendritic cells that are involved in establishing an immunosuppressive PC microenvironment [50]. For example, miR-203 is overexpressed in PC-derived exosomes and inhibits the expression of toll-like receptor 4 (TLR-4), tumor necrosis factor α (TNF-α), and interleukin 12 (IL-12) in dendritic cells, thus inducing immune tolerance [49]. In dendritic cells, the regulatory factor X-associated protein (RFXAP), a key transcription factor of the MHC II gene, is inhibited by exosomal miR-212-3p derived from PC-secreted exosomes. This decreased MHC II expression in dendritic cells boosts the establishment of an immunotolerant PC microenvironment [50]. Recently, Shen et al. showed that PC-derived exosomes induce T lymphocyte apoptosis through p38 MAPK-mediated endoplasmic reticulum stress, causing impairment of the immune response [51].

Overall, PC cells adopt several strategies to suppress host immune response, to escape from immune defenses, and to facilitate tumor growth and development. In this regard, cancer-derived exosomes contribute to the impairment of immune system surveillance functions, acting as mediators of short- and long-range intercellular communications.

### 3.3. Induction of PC Chemoresistance

In the last 10 years, both combination chemotherapy regimens and gemcitabine monotherapy in patients with a suboptimal performance status has provided unsatisfactory improvement in the survival of PC patients [80]. In addition, the drug resistance of cancer cells remains a great obstacle to successful chemotherapy. Exosome-induced chemoresistance has been recognized as a novel mechanism of drug resistance [81]. Mechanistically, exosomes take part in chemoresistance by directing drug export, affecting drug efflux pumps and the cell–cell exchange of miRNAs that contribute to drug resistance (Table 1). MicroRNA exchange via exosomes between drug-resistant and drug-sensitive tumor cells can generate a more complex chemotherapeutic heterogeneity [81]. Moreover, functional delivery of exosomal miRNAs between the tumor microenvironment and cancer cells might also promote chemoresistance [81].

The exosomal fraction of conditioned media from gemcitabine-treated PC cells (Gem-Exo) reduces PC cell chemosensitivity to gemcitabine [53]. Gene expression analyses in Gem-Exo-treated cells revealed upregulation of ROS detoxification enzymes, such as catalase (CAT) and superoxide dismutase 2 (SOD2), and downregulation of deoxycytidine kinase DCK (gemcitabine-metabolizing gene). DCK downregulation occurs through exosomal miR-155, since both miR-155 suppression or DCK restoration leads to abrogation of PC chemoresistance mediated by Gem-Exo [53]. Mikamori et al. showed that long-term exposure to gemcitabine increases miR-155 expression in PC cells, which in turn induces exosome secretion and chemoresistance through antiapoptotic effects [54]. Recently, it was also reported that exosomes secreted by gemcitabine-resistant PC cancer stem cells enhanced drug resistance in gemcitabine-sensitive PC cells by delivering miR-210, which in turn inhibits cell cycle arrest and apoptosis induced by gemcitabine and promotes tube formation and cell migration [55]. This leads to an exosome-mediated increase in the invasive and metastatic potential of cells treated with gemcitabine.

In addition to PC cell-derived Gem-Exo, CAFs, which are insensitive to gemcitabine, also contribute to drug resistance by the release of exosomes enriched in Snail and miR-146a that promote chemoresistance, EMT, and metastasis [52]. Notably, in vitro suppression of CAF exosome secretion by GW4869 decreases Snail expression in epithelial cancer cells and hampers the survival of chemoresistant PC cells [52]. These findings indicate the potential clinical relevance of associating exosome release inhibitors and chemotherapy to overcome PC gemcitabine resistance.

Fan et al. showed that exosomes from gemcitabine-resistant PANC-1 cells increase gemcitabine resistance of Mia PaCa-2 and BxPC-3 PC cells, which are otherwise sensitive to this drug. Proteomic analysis revealed that PANC-1-derived exosomes, as compared to exosomes derived from the gemcitabine-sensitive PC cell lines, overexpressed ephrin type-A receptor 2 (EphA2). In line with an important role of this receptor in chemoresistance, EphA2 knockdown in PANC-1 cells suppresses their ability to transfer exosome-mediated chemoresistance [56]. Notably, soluble EphA2 is not able to promote chemoresistance, indicating that exosomes vehiculating membrane-bound EphA2 play a fundamental role in chemoresistance induction [56].

It was also shown that the GAIP-interacting protein C terminus (GIPC) is involved in PC drug resistance. In this regard, PC cells depleted of GIPC become more sensitive to gemcitabine [28]. Notably, proteomic analysis of exosomes isolated from GIPC-deficient PC cells revealed a significant enrichment in proteins involved in drug resistance. In particular, the drug-resistant ATP-binding cassette sub-family G member 2 (ABCG2) protein was markedly overexpressed, suggesting its potential segregation in vesicles and, consequently, its ineffectiveness in mediating chemoresistance [28].

Overall, several mechanisms related to exosome-mediated induction of drug resistance in PC have been elucidated. However, further studies are needed to better understand how exosomes can mediate and transfer chemoresistance in PC and how they could be targeted to improve therapeutic options in this highly chemoresistant tumor.

## 4. Role of PC-Derived Exosomes in the Pathogenesis of Diabetes and Weight Loss

As mentioned above, exosomes derived from tumor and non-tumor cells may play an important role in modulating metabolic activity at distant sites. One of these PC effects on metabolic activity has been linked to the pathogenesis of diabetes mellitus. PC is associated with diabetes mellitus and this evidence is supported by the fact that not only is there a very high prevalence of diabetes in PC patients, but also by a close temporal relationship between PC diagnosis and the onset of diabetes [82]. Several lines of evidence indicate that exosomes play an important role in this association. For instance, Javeed and colleagues showed that adrenomedullin (AM) is delivered from PC to β cells as exosomal cargo. These AM-positive exosomes enter β cells through caveolin-mediated endocytosis or macropinocytosis, causing impaired insulin secretion, upregulation of ER stress genes, and increased reactive oxygen/nitrogen species [83]. Interestingly, the blockade of the interaction between AM and its receptors abrogates the inhibitory effect of PC-derived AM-positive exosomes on insulin secretion [83].

Other putative mechanisms responsible for the association between PC and new-onset diabetes mellitus involve the induction of insulin resistance. To explore these mechanisms, the effects of exosomes released both by murine PC and by ductal epithelial cells on murine skeletal muscle cells were analyzed [84]. The study showed that exosomes derived from PC cells entered skeletal muscle cells and induced a state of insulin resistance by inhibition of glucose transport and promotion of lipidosis. It was also shown that PC-derived exosomes could inhibit insulin and PI3K/Akt/FoxO1 signaling pathways, thereby impairing Glut4 translocation and glucose transport [84]. Exosomal miRNAs were implicated in this process. In particular, miR-666-3p, miR-540-3p, miR-125b-5p, and miR-450b-3p promoted FoxO1 expression, a critical player in muscle insulin resistance, while miR-883b-5p, miR-666-3p, miR-450b-3p, and miR-151-3p were involved in the downregulation of Glut4 expression [84]. These findings are in line with the theories of “metabolic reprogramming” and “metabolic crosstalk” in cancer [84]. Furthermore, PC patients diagnosed with new-onset diabetes were recently shown to produce significantly reduced levels of glucose-dependent insulinotropic peptide (GIP), which is secreted mainly by enteroendocrine cells [85]. It was shown that PC-derived exosomes inhibit insulin secretion by reducing the levels of GIP and glucagon-like peptide-1 (GLP-1) through decreased expression of proprotein convertase subtilisin/kexin type 1/3 (PCSK1/3). Differentially expressed exosomal miRNAs (miR-6796-3p, miR-6763-5p, miR-4750-3p, and miR-197-3p) suppressed the expression of PCSK1/3 and were identified to be responsible for the inhibitory effects on GIP and GLP-1 production [85]. Interestingly, PC-derived exosomes are transported via pancreatic juice rather than blood to target GIP and GLP-1, producing cells into the gut [85].

In addition to diabetes, weight loss also occurs in conjunction with PC diagnosis and exosomes appear to play a crucial role in this process [58]. It was found that exosomes secreted from PC induce lipolysis in subcutaneous adipose tissue and exosomal AM is a candidate mediator of this effect [58]. Both AM and PC-derived exosomes promoted lipolysis in murine and human adipocytes, which was abrogated by AM receptor blockade. Mechanistically, adrenomedullin stimulates lipolysis by activation of the MAP kinase/ERK pathways and by hormone-sensitive lipase phosphorylation [58].

Overall, both new-onset diabetes mellitus and weight loss arise several months before the clinical presentation of PC. They appear as paraneoplastic phenomena characterized by metabolic changes related to tumor-secreted molecules, also delivered by exosomes, and represent potential clues for early diagnosis of PC.

## 5. Exosomes as Biomarkers for Diagnosis and Prognosis of Pancreatic Cancer

PC diagnosis is very often delayed by the lack of specific symptoms at early stages of the disease. Thus, most patients at diagnosis are already affected by locally advanced or metastatic disease, which is resistant to current treatments, resulting in a very poor prognosis [1]. Commonly used imaging techniques, such as computed tomography and endoscopic ultrasound, are unsatisfactory for PC screening [25]. Serum carbohydrate antigen (CA19-9) is the only FDA-approved biomarker for PC diagnosis, albeit with low sensitivity and specificity (70–90% and 68–91%, respectively) [16]. Hence, it is necessary to find novel robust tumor biomarkers for early PC diagnosis. Considering exosomes’ stability and their abundance in various biological fluids, exosomal miRNAs and proteins are amongthe candidates in the search for novel biomarkers (Table 2) [16].

**Table 2:** Exosomes as diagnostic and prognostic biomarkers for pancreatic cancer.

Biomarker Type	Exosomal Marker	Sample Size	Clinical Significance	Ref.
Diagnostic	ZIP4 (serum)	70 (24 PCs vs. 46 HCs)	Discrimination between PCs and healthy controls	[44]
	miR-17-5p, miR-21 (serum)	49 (22 PCs vs. 27 non-PCs/HCs)	Discrimination between PC and non-PC patients (sensitivity 72.7% and specificity 92.6% for miR-17-5p); sensitivity 95.5% and specificity 81.5% for miR-21); high levels of miR-17-5p significantly correlate with advanced PCs	[86]
	miR-21, miR-155, miR-31, let-7a, miR-221, miR-181a, miR-935, miR-508 (plasma)	60 (40 PCs/CPs vs. 20 HCs)	Discrimination between PCs/CPs and healthy controls	[87]
	miR-10b, miR-21, miR-30c, miR-181a, miR-let7a (plasma)	46 (29 PCs vs. 17 CPs/HCs)	MicroRNA signature discriminating between PCs and CPs/healthy controls, sensitivity and specificity of 100% for all biomarkers	[88]
	miR-10b (plasma)	9 (3 PCs vs. 6 CPs/HCs)	Discrimination between PCs and CPs/healthy controls	[89]
	miR-196a, miR-1246 (plasma)	30 (15 PCs vs. 15 HCs)	Discrimination between PCs and healthy controls	[90]
	miR-3940-5p/miR-8069 (urine)	80 (43 PCs vs. 37 CPs/HCs)	Discrimination between PCs and CPs/healthy controls; exosomal miRNA ratio higher in urine than in sera of PC patients	[91]
	Glypican-1 (GPC1) (serum)	290 (190 PC vs. 100 HCs)	Discrimination between PCs and healthy controls or benign pancreatic disease, sensitivity and specificity of 100%	[92]
	Glypican-1 (GPC1) (serum)	43 (22 PCs vs. 21 non-PCs/HCs)	Discrimination between PCs, healthy controls or benign pancreatic disease, sensitivity 81% and specificity 52%	[93]
	PDAC^EV^ signature (EGFR, EPCAM, MUC1, GPC1, WNT2) (plasma)	43 (22 PCs vs. 21 non-PCs/HCs)	Discrimination between PCs, healthy controls or benign pancreatic disease, sensitivity 86% and specificity 81%	[93]
	CKAP4 (serum)	85 (47 PCs vs. 38 non-PCs/HCs)	Discrimination between PC patients IHC+ for CKAP4 and PC patients IHC- for CKAP4, HCs or non-PC patients	[94]
Prognostic	miR-3607-3p (plasma)	60 (40 PCs vs. 20 HCs)	Low levels predict poor prognosis in PC patients	[34]
	miR-301a-3p (serum)	62 (50 PCs vs. 12 HCs)	High levels predict poor prognosis in PC patients	[40]
	Sox2ot (plasma)	40 (20 PCs vs. 20 HCs)	High levels correlate with TNM stage and poor overall survival in PC patients	[41]
	circ-PDE8A (plasma)	113 (93 PCs vs. 20 non-PCs)	High levels correlate with TNM stage and poor overall survival in PC patients	[42]
	circ-IARS (plasma)	40 (20 metastatic PCs vs. 20 non-metastatic PCs)	High levels correlate with TNM stage and overall survival in PC patients	[43]
	MIF (plasma)	55 (40 metastatic/non-metastatic PCs vs. 15 HCs)	High levels correlate with progression of disease post-diagnosis and prediction of liver metastasis	[45]
	miR-451a (plasma)	70 (50 stage I/II PCs vs. 20 HCs)	High levels predict recurrence and poor prognosis in PC patients	[95]
	Glypican-1 (GPC1) (serum)	290 (190 PC vs. 100 HCs)	High levels of GPC1^+^ exosomes correlate with tumor burden and reduced survival of PC patients	[92]
	Glypican-1 (GPC1) (serum)	59 (27 PC vs. 32 non-PCs)	High levels of GPC1^+^ exosomes correlate with tumor size	[96]
	c-Met, PDL-1 (serum)	91 (55 PCs vs. 36 non-PCs)	High levels after surgery predict poor survival for PC patients	[97]

**Abbreviations**: Zinc transporter protein 4, ZIP4; Pancreatic cancers, PCs; Healthy controls, HCs; Chronic pancreatitis, CPs; Epidermal growth factor receptor, EGFR; Epithelial cell adhesion molecule, EPCAM; Mucin 1, MUC1; Wingless-type MMTV integration site family, member 2, WNT2; Cytoskeleton-associated protein 4, CKAP4; Immunohistochemistry, IHC; Macrophage migration inhibitory factor, MIF; Proto-oncogene mesenchymal-epithelial transition factor, c-Met; Programmed death-ligand 1, PD-L1.

A study identified higher levels of exosomal miR-17-5p and miR-21 in the sera of PC patients as compared to healthy controls and to non-PC patients with benign pancreatic tumors, ampullary carcinomas, or chronic pancreatitis (CP) [86]. Moreover, high levels of miR-17-5p were significantly correlated with metastasis and advanced PC, supporting the value of this miRNA as a potential biomarker for unresectable tumors [86]. Differently, miR-21 was not significantly correlated with PC differentiation and tumor stage [86]. In a more recent study, differential expression of eight miRNAs (i.e., miR-21, miR-155, miR-31, miR-let-7a, miR-221, miR-181a, miR-935, miR-508) was found in the plasma of patients with CP and PC patients, as compared to healthy subjects [87]. Accordingly, another study indicated that high levels of exosomal miR-21, but also of miR-10b, miR-30c, and miR-181a, together with low levels of miR-let7a, differentiated among healthy controls, and CP and PC samples [88]. Another study confirmed that miR-10b, along with miR-196a and miR-1246, displayed increased levels in exosomes isolated from the plasma of PC patients, as compared to CP or healthy controls, thus indicating that they may represent valuable diagnostic biomarkers in PC diagnosis [89,90]. Differently, exosomal miR-451a showed a significant association with tumor size and staging in PC patients, representing a useful prognostic biomarker in predicting PC recurrence and survival [95]. Recently, Yoshizawa et al. showed that the miR-3940-5p/miR-8069 ratio in urine exosomes was elevated and specific in PC patients and this ratio tended to be higher in the urine as compared with the sera of PC patients, suggesting that it may be a potent diagnostic tool for PC [91].

Additionally, exosomal proteins play a relevant role in PC detection. A cell surface proteoglycan, glypican-1 (GPC1), is specifically enriched in cancer cell-derived exosome, and it is significantly elevated in PC patients [92]. Exosomal GPC1+ levels represent a biomarker for all stages of PC and were shown to have 100% sensitivity and specificity in PC detection [92]. GPC1+ circulating exosomes allow patients with PC to be distinguished from those with benign pancreatic disease or healthy individuals, while serum CA 19–9 levels fail in this stratification [92]. Moreover, the GPC1+ exosome level is indicative of tumor stage and distant metastases, and its low levels are related to increased survival [92]. However, other studies showed controversial results on GPC1+ exosomes as powerful diagnostic tools for PC [93,96]. In this regard, Yang et al. [93] showed that single GPC1 screening had lower sensitivity (82%) and specificity (52%) than in the study of Melo et al. [92] and identified an EV-based protein signature that appears more robust than GPC1+ exosomes alone for PC diagnosis. In particular, a multiplexed nanoplasmonic sensor assay was used for EV phenotypic characterization in 135 patients with pancreatic pathologies [93]. A panel of tumor-derived extracellular vesicle markers, including EGFR, EPCAM, HER2, MUC, GPC1, WNT2, and GRP94, was investigated. The PDAC^EV^ signature calculated as the unweighted sum of EGFR, EPCAM, MUC1, GPC1, and WNT2 signals showed an 86% sensitivity and 81% specificity in distinguishing pancreatic cancer patients from healthy controls. The different experimental conditions between these two studies [92,93] may explain the discrepancies in the sensitivity and specificity of GPC1 as a single EV-related marker for PC detection. In another small series, Frampton et al. reported an association between high expression of GPC1 on circulating exosomes and pancreatic tumor burden [96], but no difference in the blood levels of GPC1+ circulating exosomes was observed between patients with PC and those with pancreatic benign disease [96].

A recent study compared the diagnostic values of exosomal ZIP4 levels in the sera of patients with PC, benign pancreatic, or biliary diseases and healthy controls, suggesting that ZIP4 might be a candidate diagnostic biomarker for PC [44]. Another study showed that cytoskeleton-associated protein 4 (CKAP4) is released by PC cells via exosomes [94]. Moreover, CKAP4 was detected in the sera of PC-bearing xenografted mice and in PC patients, whereas CKAP4 was scarcely detectable in sera from normal mice and postoperative patients [94]. Thus, CKAP4 secreted with exosomes in the serum of PC patients may also represent a novel potential biomarker for PC diagnosis [94].

Additionally, c-Met (proto-oncogene mesenchymal–epithelial transition factor) and PD-L1 (programmed cell death 1 ligand 1) were analyzed in circulating exosomes from the sera of patients with PC, chronic pancreatitis, or benign serous cystadenoma of the pancreas [97]. In exosomes isolated from PC patients, c-Met levels were significantly higher than in patients with benign disease. Moreover, c-Met-positive patients showed a shorter postoperative survival time. Similarly, PD-L1-positive patients showed a significantly shorter survival after surgery, whereas exosomal PD-L1 levels were not significantly different among patients. Thus, both c-Met- and PD-L1-positive exosomes in peripheral blood might be considered negative prognostic factors for PC, whereas only c-Met-positive exosomes appear to have diagnostic potential [97].

It is worth noting that exosome-based screening for PC has several advantages as compared with traditional techniques, including the lack of invasiveness and the potential to accurately define the cellular origin for exosomal biomarkers, more than with other circulating biomarkers. Thus, although additional clinical studies are needed for further validation, current evidence indicates that exosomes in liquid biopsies, urine, or other body fluids might represent novel promising biomarkers for PC diagnosis and prognosis.

## 6. PC Therapy: Exosomes as Drug Carriers and Therapeutic Targets

Exosomes have been reported to be putative therapeutic targets and potential drug delivery carriers in PC treatment (Table 3). In this regard, due to their unique lipid bilayer structure, exosomes can be considered as nanoparticle carriers for drugs and bioactive molecules. However, as compared to other nanoparticle carriers, exosome-based therapies are likely to have no toxic side effects and low immunogenicity [98]. Both of these characteristics support the potential safety of exosomes in treating cancers.

**Table 3:** Potential applications of exosomes in pancreatic cancer treatment.

Treatment Strategy	Therapeutic Agent/Approach	Main Findings of the Study	Ref.
Inhibition of exosome biogenesis	GW4869	The exosome release inhibitor GW4869 overcomes gemcitabine-resistance associated with the increased exosome release promoted by CAFs exposed to gemcitabine and thus decreases PC cell proliferation	[52]
Inhibition of exosome secretion	SiRAB27B	In PC cells transfected with SiRAB27B, both exosome secretion and miR-155-induced gemcitabine resistance are significantly reduced	[54]
Inhibition of exosome uptake in recipient cells	REG3β	Lectin REG3β released by healthy pancreatic tissue surrounding the tumor binds to exosome surface, thereby impairing exosome uptake by recipient tumor cells, which in turn prevents PC cell metabolic changes and migration	[99]
	KRAS^G12D^-siRNA	Exosomes engineered to carry a siRNA targeting the common KRAS^G12D^ mutation drastically reduce PC growth in vivo improving survival of PC mouse models	[100]
Drug/small RNA-delivery	Paclitaxel	Mesenchymal stromal cells loaded with paclitaxel significantly reduced PC cell proliferation through exosomes released into conditioned media	[101]
	Gemcitabine	Gemcitabine-loaded exosomes enable drug uptake in PC cells, significantly increasing both concentration and cytotoxic effects of gemcitabine in vivo	[102]
Immunity enhancement	miRNA depletion	Ultrafiltered miRNA-depleted exosome lysates isolated from cultured PC cell supernatants improve tumor-killing activity of immune cells towards PC cells	[103]
	Staphylococcal enterotoxin B (SEB)	Novel structures based on protein anchorage of the potent immune stimulator SEB on exosomes promote PC cell apoptosis and might be used to stimulate immune response against PC	[104]
	Genetic manipulation of PC cells	Genetic manipulation of PC cells to induce exosomal transfer of miR-155 and miR-125b-2 to macrophages induces their reprogramming towards an antitumor M1 phenotype	[105]
	DCs loaded with PC-derived exosomes	Vaccination by DCs loaded with PC-derived exosomes improves response to chemotherapy, slows tumor growth and increases survival of PC tumor-bearing mice	[106]

**Abbreviations:** Cancer-associated fibroblasts, CAFs; Pancreatic cancer, PC; Small interfering RNA RAB27B, siRAB27B; Regenerating islet-derived 3β, REG3β; Dendritic cells, DCs.

### 6.1. Exosomes as Therapeutic Targets

Exosomes play key roles in PC progression and in the onset of cancer drug resistance. Thus, strategies that inhibit the production of cell exosomes may be potentially beneficial in PC treatment. As described above, CAF cells are intrinsically resistant to gemcitabine and can transmit chemoresistance to cancer cells through exosome release. Richards et al. reported that when exosome release from gemcitabine-exposed CAF cells is inhibited by GW4869, the survival and proliferation rates of PC cells are significantly reduced [52]. Similarly, gemcitabine resistance of PC cells, associated with miR-155 overexpression, is ameliorated by transfecting tumor cells with siRAB27B, which reduces the number of secreted exosomes [54].

In addition to blocking the secretion of exosomes, another potential therapeutic strategy is based on inhibiting the uptake of specific exosomes by recipient cells. In this regard, it was shown that release of the lectin REG3β by healthy pancreatic tissue surrounding the tumor interferes with the uptake and internalization of exosomes by recipient cells, due to the binding of this lectin to glycoproteins on the exosome surface [99]. Consequently, vesicles blocked by the presence of REG3β lose their ability to modulate several biological processes, including polarization of macrophages towards an inflammatory M1 phenotype, migration of PC cells, and typical metabolic changes of different cells within the tumor microenvironment [99]. Considering that EVs may have detrimental or advantageous effects in the biology of PC (Figure 3), the therapeutic potential of inhibiting the whole exosomal uptake by recipient cells needs to be further investigated.

Overall, suppression of exosome secretion or uptake represents a novel potential strategy to hamper PC progression and cancer drug resistance in PC therapy.

### 6.2. Exosomes as Drug Vehicles

Drug-loaded exosomes have great potential for delivering chemotherapeutics to drug-resistant PC cells. Exosomes are ideal drug carriers because they can be targeted to selected recipient cells through specific transmembrane proteins. Thus, drugs having high toxicity could be loaded in exosomes and transferred to target cells at higher concentrations, avoiding systemic toxicity [107]. In addition, among the membrane-anchored proteins, exosomes express the integrin-associated transmembrane protein CD47 [108] that protects exosomes from phagocytosis by monocytes and macrophages and increases the exosome half-life [100].

Because of their high delivery efficiency and biocompatibility, exosomes have emerged as candidates for the delivery of several chemotherapeutics or molecules with anticancer properties. Kim et al. demonstrated that the exosome-encapsulated paclitaxel can significantly increase cytotoxicity in drug-resistant cancer cells by more than 50 times [109]. Specifically, mesenchymal stromal cells loaded with paclitaxel significantly reduced PC cell proliferation through exosomes released into conditioned media [101]. Exosomes have also been used to deliver the natural phenol curcumin to recipient PC cells, promoting cytotoxicity in vitro [110]. Similarly, exosomes containing the apoptosis-inducing survivin mutant T34A increase the gemcitabine sensitivity of the MiaPaCa-2 pancreatic cancer cell line [111]. Recently, gemcitabine was packaged into autologous exosomes (ExoGEM) for PC-targeted chemotherapy [102]. In vivo, ExoGEM facilitates cellular drug uptake and contributes by significantly increasing both the concentration and cytotoxic effects of gemcitabine, while heterologous cellular uptake is less efficient [102].

In addition to common chemotherapeutics, exosomes can also be used to deliver small RNAs (i.e., siRNA and miRNA) into recipient cells, due to their natural role in intercellular RNA transport. Recently, Lamichhane et al. [112] described a sonication method that allows exosomal incorporation of many different small RNA cargos targeting pro-oncogenic mRNA. In particular, they found that exosomes loaded with therapeutic HER2-siRNA are able to reduce the expression of the HER2 receptor in recipient HEK293T cells [112]. KRAS mutations are very common in PC, and tumor growth is suppressed and overall survival is improved in mouse models of PC by exosomal delivery of engineered KRASG12D siRNA or short hairpin RNA [100].

Collectively, these results indicate an interesting potential utility of exosomes as candidates for drug or small RNA cargo delivery that could be used to develop innovative therapeutic strategies for PC.

### 6.3. Exosomes as Tumor-Associated Immunity Enhancers

It is well known that immune checkpoints expressed by cancer and antigen-presenting cells (APC), such as PD-L1 and CD80/CD86, respectively, interacting with PD-1 and CTLA-4 expressed on the T cell surface, trigger negative signals, leading to immune suppression in several solid tumors, in which subsequent treatment regimens based on immune checkpoint inhibitors (ICIs) have proved to be beneficial [113]. Unfortunately, although multiple studies showed that high PD-L1 expression also detected in PC is associated with poor outcomes, suggesting that targeting PD-1/PD-L1 interaction may have therapeutic benefit in these patients [11,113], promising preclinical findings have not translated into clinical success when ICIs were tested as single agents in PC patients [114,115]. Modest results were also obtained in PC patients with defective mismatch repair, which ideally would make them suitable for ICI-based treatments due to the elevated immunogenicity of MMR-deficient tumors [116]. The complex cross-talk between PC cells and microenvironment components is likely to be one of the most critical features that at least partly explains immunotherapy failure in the treatment of this disease. In this regard, the presence of inhibitory cytokines, immunosuppressive cells, and hypoxia, together with a dense fibrotic stroma, create a powerful immunosuppressive PC environment that hampers substantial responses to immunotherapy.

In such a scenario, exosomes might be exploited in cancer immunotherapy based on their involvement in the modulation of immune responses. On the one hand, exosomes derived from immune cells, such as mature dendritic cells, M1-polarized macrophages, and NK cells, may activate an immune response against different tumors, counteracting the immune-suppressive tumor microenvironment [14,117,118]. On the other hand, recent evidence obtained in mouse models of different tumor types indicates that tumor-derived exosomal PD-L1 checkpoint molecules may act systemically to suppress an antitumor immune response and that this suppression may be reversed by interfering with exosomal PD-L1 release [119].

A few studies investigated the potential of exosome manipulations as antitumor immunity enhancers in PC. In this regard, one study, considering that tumor-derived exosomes can inhibit the immune response by transferring several miRNAs to immune cells, whereas exosomal proteins may increase the immune response, tested the effects of ultrafiltered and miRNA-depleted exosome lysates (UELs) derived from PC cells [103]. These UELs increased the tumor-killing capacity of dendritic cells/cytokine-induced killer cells towards PC cells [103], suggesting that miRNA-depleted exosomes derived from PC cells might represent valuable immunotherapeutic tools in PC. Another study reported that PC-derived exosomes can be packaged with staphylococcal enterotoxin B, which is a potent immune stimulator [120] that is able to stimulate T cell activation [104], suggesting that such a strategy might be used to stimulate an immune response against PC. It was also shown that the presence of heat shock protein 70 (Hsp70) on the surface of PC-derived exosomes stimulates NK cytolytic activity against PC cells [121], suggesting the induction of Hsp70 expression on the surface of PC exosomes might be a useful strategy to enhance the immune response. More recently, genetic manipulation of PC cells to induce exosomal transfer of miR-155 and miR-125b-2 to macrophages induces their reprogramming from a tumor-promoting and immunosuppressive M2 phenotype to an antitumor M1 phenotype [105], suggesting that a similar approach might be beneficial in the treatment of PC. A recent study showed that PC exosomes expose several tumor-associated antigens that bind circulating autoantibodies, thereby exerting a decoy function [122]. This, in turn, hampers complement-dependent cytotoxicity and possibly antibody-dependent cell-mediated cytotoxicity against PC, suggesting that inhibition of PC exosome biogenesis/secretion or selective depletion of circulating PC exosomes by affinity capture may be a useful strategy to enhance the antitumor immune response [122]. An additional study showed that vaccination, using dendritic cells loaded with exosomes derived from PC cells, improved the response to chemotherapy, slowing tumor growth and increasing the survival of PC tumor-bearing mice [106].

Overall, studies analyzing exosomes as potential immunity enhancers against PC have so far provided some preliminary but interesting results, which encourage further investigations in this field.

## 7. Future Perspectives and Limitations

In summary, exosomes derived from PC cells play a crucial role in the pathogenesis of cancer because they participate in all steps of disease development through distinct mechanisms. Because of their structure and cargo, exosomes allow cross-talk between cells within the tumor microenvironment, promoting its remodeling. This process leads to increased PC cell proliferation, survival, invasive potential, chemoresistance, and escape from immunosurveillance. In addition, PC exosomes may act at distant sites to promote metastasis through premetastatic niche formation. One of the open questions is how to activate the immune response against the poorly immunogenic PC and whether exosomes can be manipulated to achieve this effect. There is a relative lack of studies on this potential application of exosomes in PC therapy. Considering that interfering with exosome-mediated immune checkpoint inhibition in other tumor models induces systemic antitumor immunity [119], this might also represent a promising field of study in PC, which is resistant to current immunotherapeutic approaches.

Based on their peculiar protein and miRNA cargoes, exosomes have been proposed as improved diagnostic and prognostic biomarkers in PC [21,92]. Intriguingly, proteome profiling of secretome and exosomes in *BRCA1*-deficient breast cancers was able to cluster most human *BRCA1-* and *BRCA2-*related breast carcinomas, yielding potential biomarkers for the early diagnosis of cancer [123]. It would be interesting to investigate whether a similar approach could be applied in the subgroup of PC patients carrying *BRCA* mutations to identify markers for non-invasive diagnosis of *BRCA*-deficient PC tumors, which might benefit from treatment with specific targeted agents. However, large-scale cohort studies with standardized exosome isolation and purification techniques appear necessary to confirm the value of the clinical application of exosomes to the diagnosis and prognosis of PC. Exosomes are also being considered as candidate therapeutic targets and drug delivery carriers for PC [54,102]. Because of their lipid bilayer structure, no toxic side effects, biological barrier permeability, low immunogenicity, and biocompatibility, exosomes represent nanoparticle carriers that might be employed to transport drugs and bioactive molecules [13].

In conclusion, the study of exosomes in PC is a very promising field of research with multiple potential applications, and further investigations are needed for clinical translation of this research.
